# Functional analyses of the NRT2 family of nitrate transporters in *Arabidopsis*

**DOI:** 10.3389/fpls.2024.1351998

**Published:** 2024-03-04

**Authors:** Na Xu, Li Cheng, Yuan Kong, Guiling Chen, Lufei Zhao, Fei Liu

**Affiliations:** ^1^ School of Biological Science, Jining Medical University, Rizhao, Shandong, China; ^2^ Agricultural Science and Engineering School, Liaocheng University, Liaocheng, Shandong, China

**Keywords:** *Arabidopsis*, high-affinity nitrate transport system (HATS), NRT2s, C-N homeostasis, plant-microbe interactions, systemic nitrate signaling

## Abstract

Nitrogen is an essential macronutrient for plant growth and development. Nitrate is the major form of nitrogen acquired by most crops and also serves as a vital signaling molecule. Nitrate is absorbed from the soil into root cells usually by the low-affinity NRT1 NO_3_^-^ transporters and high-affinity NRT2 NO_3_^-^ transporters, with NRT2s serving to absorb NO_3_^-^ under NO_3_^–^limiting conditions. Seven NRT2 members have been identified in *Arabidopsis*, and they have been shown to be involved in various biological processes. In this review, we summarize the spatiotemporal expression patterns, localization, and biotic and abiotic responses of these transporters with a focus on recent advances in the current understanding of the functions of the seven *AtNRT2* genes. This review offers beneficial insight into the mechanisms by which plants adapt to changing environmental conditions and provides a theoretical basis for crop research in the near future.

## 1 Introduction

Plants rely on nitrogen (N) as an essential macronutrient that is vital for their growth and productivity. Nitrate (NO_3_^-^) is the most abundant source of inorganic nitrogen taken up by most terrestrial plant species (Crawford and Forde, 2002). Kinetic criteria have been used to characterize such nitrate uptake as being mediated by three distinct systems, including a low-affinity transport system (LATS) as well as inducible and constitutive high-affinity transport systems (iHATS and cHATS) (Glass et al., 1995; Crawford and Glass, 1998; Forde, 2000). The cHATS and iHATS systems are generally active at NO_3_^-^ concentrations in the 10-250 μM range, whereas LATS activity is only apparent when these concentrations exceed 250 μM. The NO_3_^-^ affinity of the iHATS (Km: 13-79 μM) is lower than that of the cHATS (Km: 6-20 μM) (Forde and Clarkson, 1999), iHATS capacity for the uptake of NO_3_^-^ greatly exceeds that of cHATS. For example, analyses of iHATS activity in response to induction with a NO_3_^-^ concentration of 100 μM yielded a V_max_ that was roughly 25-fold higher than that for the cHATS (Siddiqi et al., 1990). Members of the NRT1 protein family serve as low-affinity NO_3_^-^ transporters with Km values in the mM range, while members of the NRT2 protein family function as high-affinity transporters with Km values in the μM range (Wang et al., 2012; O'Brien et al., 2016).The *Aspergillus nidulans crnA* gene was the first high-affinity NO_3_^-^ transporter cloned from a eukaryotic species, and mutations in this gene can confer chlorate (ClO_3_^-^) resistance while resulting in the partial impairment of NO_3_^-^ uptake (Brownlee and Arst, 1983; Unkles et al., 1991). The uptake of NO_2_^-^ and NO_3_^-^ in *Chlamydomonas reinhardtii* was subsequently determined to be under the control of three genes related to *crnA*, including *CrNRT2.1*, *CrNRT2.2*, and *CrNRT2.3* (Quesada et al., 1994; Galván et al., 1996; Quesada et al., 1998). More recently, researchers have identified high-affinity nitrate transporters from a wide range of plants including *Arabidopsis thaliana*, *Hordeum vulgare*, and *Nicotiana plumbaginifolia* (Quesada et al., 1997; Filleur and Daniel-Vedele, 1999; Zhuo et al., 1999; Vidmar et al., 2000). In the case of *Arabidopsis*, RT-PCR analyses performed using degenerate primers led to the initial cloning of the two related *AtNRT2.1* and *AtNRT2.2* transporter genes (Zhuo et al., 1999). With the completion of the *Arabidopsis* genome project, a total of seven *Arabidopsis* NRT2 family members were identified in this model species. The roots are the predominant site of expression for all *AtNRT2* genes other than *AtNRT2.7*, which is expressed at the highest levels in seeds (Orsel et al., 2002; Okamoto et al., 2003; Chopin et al., 2007a). The responsivity of different *NRT2* genes to changes in nitrate availability following N starvation varies markedly. For example, NO_3_^-^ exposure strongly induced the expression of *NRT2.1* and *NRT2.2*, while *NRT2.4* was modestly upregulated, *NRT2.5* expression was repressed, and the expression of *NRT2.3*, *NRT2.6*, and *NRT2.7* was unaffected by the available nitrate supply (Okamoto et al., 2003). In addition to their roles as mediators of high-affinity NO_3_^-^ influx, these NRT2 family members have been reported to play key roles in an array of biological processes involved in regulating processes such as N starvation, root architecture, seed development, cadmium uptake, plant-microbe interactions, systemic nitrate signaling, and the maintenance of appropriate nitrogen and carbon homeostasis (**Table 1**). This review offers an overview of information that is currently known regarding the molecular mechanisms and functions associated with plant members of the *NRT2* gene family.

**Table 1:** Summary of *Arabidopsis NRT2* nitrate transporter genes.

Name	Locus	Spatial Expression Pattern	Protein Localization	Nitrate Response	Other Regulations	Interaction With NRT3.1	Functions in *Arabidopsis*
NRT2.1	At1G08090	Mainly expressed in the roots, especially in the epidermal, cortical, and endodermal cell layers of the mature root parts	Plasma membrane	Induction	N starvation induction, ammonium and glutamine repression, light and sugar induction, cadmium repression	Yes	High-affinity nitrate uptake, cadmium uptake, plant-microbe interactions, systemic N signal, carbon and nitrogen metabolism
NRT2.2	At1G08100	Expressed in roots at low levels	Plasma membrane	Induction	Cadmium repression	Yes	High-affinity nitrate uptake
NRT2.3	At5G60780	Expressed in roots and shoots	Plasma membrane	Constitutive	Not known	Yes	Not known
NRT2.4	At5G60770	Expressed in the lateral root epidermis and the shoot vascular tissue	Plasma membrane	Induction	N starvation induction, cadmium repression	Yes	High-affinity nitrate uptake, N starvation, N remobilization
NRT2.5	At1G12940	Expressed in the root hair zone of the primary and the lateral roots and in the higher-order veins of leaves	Plasma membrane	Repression	N starvation induction, PGPR strain STM196 induction	Yes	High-affinity nitrate uptake, N starvation, N remobilization, plant-microbe interactions
NRT2.6	At3G45060	Strong preferential expression in roots	Plasma membrane	Constitutive	PGPR strain STM196 induction, bacterium *Erwinia amylovora* induction	Yes	Plant-microbe interactions
NRT2.7	At5G14570	Highly expressed in seeds	Tonoplast	Constitutive	Not known	No	Seed nitrate storage, seed germination, seed color

## 2 High-affinity nitrate absorption

To date, seven NRT2 proteins have been identified in *Arabidopsis thaliana* (Glass et al., 2001). Of these, high-affinity root NO_3_^-^ influx is only mediated by NRT2.1, NRT2.2, NRT2.4, and NRT2.5, each of which exhibits distinct context-dependent contributions to this absorptive process (Filleur et al., 2001; Kiba et al., 2012; Lezhneva et al., 2014).

### 2.1 AtNRT2.1 and AtNRT2.2

The ability of *AtNRT2.1* and *AtNRT2.2* to function in an iHATS is supported by the fact that the *Atnrt2* T-DNA mutant, in which both of these genes are disrupted, exhibited a reduction in high-affinity nitrate uptake (Cerezo et al., 2001; Filleur et al., 2001). Notably, *AtNRT2.1* transcript levels are strictly correlated with high-affinity uptake of nitrogen when nitrate is supplied to plants that were initially nitrate-deprived (Okamoto et al., 2003). Further studies have explored the iHATS, cHATS, and LATS systems in *Atnrt2.1* mutant plants that were cultivated for 4 weeks in a 1 mM NH_4_NO_3_ solution followed by a 7-day nitrogen deprivation period in order to deplete nitrogen reserves (Li et al., 2007). Upon initial exposure of these plants to 100 μM ^13^NO_3_^-^, cHATS flux is first observed. Moreover, after the 1-week nitrogen deprivation period, plants were treated for 6 h with 1 mM KNO_3_ followed by exposure to 100 μM ^13^NO_3_^-^, with the resultant flux representing the combination of iHATS and cHATS flux. These two flux measurements can be used to estimate iHATS activity based on the difference between the two. These sample plants were also utilized to assess LATS influx after a 6-hour induction period with 1 mM KNO_3_ and subsequent exposure to 10 mM ^13^NO_3_^-^. These analyses revealed a ~72% reduction in iHATS activity in *Atnrt2.1* mutants without any corresponding change in cHATS or LATS flux (Li et al., 2007). Similarly, a 19% drop in iHATS flux was observed in *Atnrt2.2* mutants, whereas cHATS and LATS fluxes remained intact (Li et al., 2007). *AtNRT2.2* expression levels are significantly lower than *AtNRT2.1* levels (Zhuo et al., 1999; Orsel et al., 2002; Okamoto et al., 2003), but these levels were ~3-fold higher in *Atnrt2.1* mutant plants, indicating that *AtNRT2.2* overexpression may partially compensate for the loss of *Atnrt2.1.* Consistent with such a mechanism, *nrt2.1 nrt2.2* double mutants exhibit more dramatic iHATS and cHATS fluxes by ~80% and 30%, respectively, relative to *nrt2.1* and *nrt2.2* single mutants. These data emphasize the importance of *NRT2.1* as the key driver of iHATS activity, whereas *NRT2.2* exhibits a smaller compensatory role in this context (Li et al., 2007).

*AtNRT2.1* expression is primarily evident in roots (Orsel et al., 2002), and it primarily localizes to the plasma membrane of root epidermal and cortical cells, consistent with this being the primary site of nitrate uptake (Wirth et al., 2007; Chopin et al., 2007b). NRT2.1 protein level changes reportedly differ from corresponding shifts in the mRNA expression of *NRT2.1* (Wirth et al., 2007), and *35S::NRT2.1* transformants constitutively overexpressing *NRT2.1* still exhibited reductions in HATS activity (Laugier et al., 2012), consistent with mechanisms responsible for post-translationally regulating *NRT2.1*. Phosphoproteomic analyses indicated that NRT2.1 is subject to phosphorylation, with the degree of its phosphorylation shifting as a function of the availability of nitrate (Engelsberger and Schulze, 2012; Menz et al., 2016). NRT2.1 reportedly harbors four phosphorylation sites as confirmed through high-accuracy mass spectrometry-based efforts to detect phosphopeptides (Engelsberger and Schulze, 2012; Menz et al., 2016; Jacquot et al., 2020). The Ser28 phosphorylation of NRT2.1 is evident in plants subject to N starvation, but dephosphorylation occurs rapidly when nitrate becomes available (Engelsberger and Schulze, 2012). Consistent with this observation, other studies have confirmed the stabilization and enhanced Ser28 phosphorylation of NRT2.1 under conditions of nitrate limitation. To explore the role of Ser28 phosphorylation, researchers established transgenic NRT2.1^S28E^ and NRT2.1^S28A^ plants that respectively mimic the phosphorylates and dephosphorylated forms of this protein (Zou et al., 2020). The Ser28 alanine substitution was associated with NRT2.1 destabilization, and NRT2.1^S28A^ overexpression under conditions of limited nitrate availability failed to rescue defective *nrt2* mutant plant phonotypes. In contrast, greater levels of the NRT2.1^S28E^ isoform enhanced protein stability and were sufficient to restore *nrt2* mutant phonotypes when cultivated in the presence of low nitrate levels (Zou et al., 2020). NRT2.1 Ser28 phosphorylation thus plays a key role in regulating NRT2.1 stability. Jacquot et al. further determined that the C-terminal portion of NRT2.1 (aa 494-513) is essential for the appropriate function of this protein, as demonstrated using transgenic *nrt2.1-2* mutant plants expressing truncated NRT2.1 isoforms (*NRT2.1*ΔC_494-530_ and *NRT2.1*ΔC_514-530_) (Jacquot et al., 2020). While the *pNRT2.1*::*NRT2.1*ΔC_494-530_ transgene was unable to restore HAST activity and growth to wild-type levels, the *pNRT2.1*::*NRT2.1*ΔC_514-530_ transgene was able to do so. Through mass spectrometry-based phosphopeptide detection efforts, the authors were able to identify the Ser501 phosphorylation site within this region of the protein, and the phonotypes of phosphomimetic S501D transgenic plants were comparable to those of *nrt2* mutants. Higher levels of Ser501 phosphorylation were observed under cultivation on 1 mM NO_3_^-^ followed by transfer for 4 h onto 10 mM NH_4_NO_3_, consistent with a reduction in the influx of nitrate evident in wild-type plants (Jacquot et al., 2020). Ser501 phosphorylation is thus capable of inactivating the activity of NRT2.1. Notably, this Ser501 phosphorylation site is highly conserved across plant species, emphasizing the key role that it plays as a regulator of NRT2.1 functionality (Jacquot et al., 2020). This protein has also been shown to harbor N-terminal Ser11 and C-terminal Thr521 phosphorylation sites (Menz et al., 2016), although additional research will be necessary to clarify their functions.

### 2.2 AtNRT2.4 and AtNRT2.5

AtNRT2.4 is a high-affinity nitrate transporter as demonstrated by its expression in plants and heterologous expression in *Xenopus laevis* oocytes. When *nrt2.1 nrt2.2* double mutant plants exhibiting impaired high-affinity uptake of nitrate were transformed with *NRT2.4* cDNA under the control of the root-specific RolD promoter (Fraisier et al., 2000), *NRT2.4* overexpression was associated with a pronounced increase in ^15^NO_3_^-^ uptake relative to non-transformed *nrt2.1 ntr2.2* double mutant plants under conditions of low nitrate availability (0.2 mM NO_3_^-^), supporting the ability of *NRT2.4* to regulate the high-affinity uptake of NO_3_ (Kiba et al., 2012). To further confirm its ability to function in this regulatory context, Xenopus oocytes were injected for 3 days with *NRT2.4* mRNA or with water as a vehicle control, followed by exposure for 16 h to 0.2 mM Na^15^NO_3_. Subsequent analyses of the accumulation of ^15^N within oocytes revealed that those oocytes injected with the *NRT2.4* mRNA-injected oocytes took up significantly more NO_3_^-^ than water-injected controls (Kiba et al., 2012).

*AtNRT2.4* levels in plant roots were lower than those of *AtNRT2.1* at baseline, but it is strongly upregulated in response to N deprivation. When growing plants on complete N medium for 7 days followed by N starvation for 5 days, wild-type plants exhibited maximal *NRT2.4* expression. Significantly decreased ^15^NO_3_^-^ uptake relative to wild-type was detected in *nrt2.4* null mutants supplied with extremely low concentrations of ^15^NO_3_^-^ (0.025 or 0.01 mM), whereas no differences between the two were apparent when the available concentration of ^15^NO_3_^-^ was higher (0.2, 0.5, or 6 mM). This highlights a role for NRT2.4 as a mediator of very-high-affinity NO_3_^-^ uptake. Much like *NRT2.4*, the transformation of *nrt2.1 ntr2.2* double mutants with *NRT2.5* under the control of the RolD promoter resulted in a pronounced increase in the influx of ^15^NO_3_^-^ in roots as compared to non-transformed double mutants in the presence of 0.2 mM NO_3_^-^ conditions, consistent with the ability of NRT2.5 to serve as a NO_3_^-^ transporter (Lezhneva et al., 2014). In contrast to *nrt2.4* mutants for which no alterations in ^15^NO_3_^-^ influx were evident relative to wild-type plants, *nrt2.5* mutants exhibited significantly reduced high-affinity ^15^NO_3_^-^ influx in the presence of 0.2 mM NO_3_^-^ (Lezhneva et al., 2014). NRT2.5 therefore functions as a high-affinity transporter of nitrate.

### 2.3 Two-component high-affinity nitrate transporters

Besides transcriptional regulation, posttranscriptional events also can influence NRT2 protein activity and/or abundance, strongly influencing HATS functionality. Early studies demonstrated that the functionality of the *C. reinhardtii* was dependent on two gene products. The genes that encode these two proteins, *CrNRT2* and *CrNAR2*, are present within a single cluster of nitrate-regulated genes, and mutant plants with deletions in this region of the genome exhibit dramatically lower levels of high-affinity nitrate uptake that were only restored by the transformation of these plants with constructs encoding *CrNAR2* and either *CrNRT2.1* or *CrNRT2.2*, whereas none of these constructs alone were sufficient (Quesada et al., 1994). Studies of Xenopus oocytes provided further confirmation of the existence of this two-component high-affinity nitrate transport system, as the injection of mRNAs *CrNAR2* or *CrNRT2.1* alone failed to induce nitrate currents, whereas high levels of nitrate uptake were evident when both were co-injected with one another (Zhou et al., 2000a). Similar findings were also detected in barley such that only the co-injection of Xenopus oocytes with the *HvNRT2.1* and *HvNAR2.3* mRNAs encoding homologous barley proteins was sufficient to enhance nitrate transport (Tong et al., 2005; Ishikawa et al., 2009).

Through subsequent research efforts, researchers determined that *Arabidopsis* also encodes a two-component high-affinity nitrate transport system. Okamoto et al. (2006) searched for genes homologous to the *NAR2* sequences from *C. reinhardtii*, ultimately leading to the identification of the *AtNRT3.1* and *AtNRT3.2* genes (Okamoto et al., 2006; Feng et al., 2011a), the former of which was expressed at much higher levels than the latter. Strong *AtNRT3.1* upregulation was evident when the roots of plants that had been N starved were treated for 3 or 6 h with 1 mM KNO_3_, whereas only limited upregulation of *AtNRT3.2* was evident at the 6 h time point (Okamoto et al., 2006). Relative to wild-type plants, *Atnrt3.1* mutants exhibited a significant reduction in root nitrate influx under conditions of low ^13^NO_3_^-^ availability (10 - 150 μM), consistent with a role for this gene product as a regulator of NO_3_^-^ HATS activity. A ~70% reduction in iHATS activity was reported for *Atntr2.1* mutants (Filleur et al., 2001; Li et al., 2007), whereas this reduction was upwards of 95% when *AtNRT3.1* was mutated (Okamoto et al., 2006; Orsel et al., 2006). Oocyte injection experiments in which the *NRT2.1* or *NRT3.1* mRNAs were individually injected or co-injected revealed that significant uptake of ^15^NO_3_^-^ was only apparent following the co-injection of both genes (Orsel et al., 2006). *Arabidopsis* HATS activity is thus dependent on both the *AtNRT2.1* and *AtNRT3.1* genes, in line with the phenotypes observed in *C. reinhardtii* (Quesada et al., 1994; Zhou et al., 2000a). These data suggest that while *NRT3.1* is dispensable for the regulation of *NRT2.1* transcription, it can serve as a facilitator of the transport activity of the NRT2.1 protein, potentially through a mechanism mediated by direct interactions. Additional yeast split-ubiquitin system assays indicated that the NRT2.1 and NRT3.1 proteins are capable of interacting with one another (Orsel et al., 2006), and this interaction localizes to the plasma membrane (Yong et al., 2010). Consistently, an absence of NRT2.1 plasma membrane localization was evident in *nrt3.1* mutants (Wirth et al., 2007). Further confirming this result, Yong et al. conducted western blotting experiments in which they used anti-NRT2.1 to detect a 150-kDa oligomeric polypeptide extracted from the root membrane fraction, and this fraction was further resolved, revealing it to be composed of NRT2.1 (48 kDa) and myc-tagged NRT3.1 (26 kDa). This, coupled with the absence of this 150-kDa complex in *nrt2.1* or *nrt3.1* mutants, suggests that a tetrameric complex composed of two NRT2.1 subunits and two NRT3.1 subunits may be responsible for high-affinity nitrate uptake activity (Yong et al., 2010). With the exception of AtNRT2.7, which was identified as a tonoplast transporter (Chopin et al., 2007a), all NRT2 family members were shown to be capable of engaging in strong interactions with NRT3.1 in bimolecular fluorescence complementation and yeast two-hybrid experiments (Kotur et al., 2012). In Xenopus oocytes, different *NRT2* mRNAs were injected alone or in combination with *NRT3.1* to evaluate the effects on nitrate uptake. These experiments revealed that *NRT3.1* and *NRT2* co-injections were associated with greater ^15^NO_3_^-^ uptake, with this effect being particularly pronounced for *NRT3.1* coinjection with *NRT2.1*/*NRT2.5*, which yielded respective increases in nitrate uptake of 532% and 334%, as compared to only slight increases when co-injected with *NRT2.3*/*NRT2.4* (Kotur et al., 2012). Much like NRT2.1, NRT2.5 was also capable of forming a 150-kDa tetrameric complex with NRT3.1 at the plasma membrane to facilitate the high-affinity uptake of nitrate (Yong et al., 2010; Kotur and Glass, 2015). The existence of two-component NRT2/NAR2 nitrate uptake machinery has also been confirmed in plants including barley (Tong et al., 2005; Ishikawa et al., 2009), rice (Yan et al., 2011), wheat (Taulemesse et al., 2015), maize (Pii et al., 2016; Liu et al., 2020), and chrysanthemum (Gu et al., 2016). This system is not universal, however, as the *NRT2.1* homolog in *A. nidulans*, *crnA*, did not require any corresponding *NAR2* activity in Xenopus oocytes to facilitate nitrate current generation (Zhou et al., 2000b). These differences may be related to the longer *crnA* central loop and the lack of any homolog of *NAR2* in *A. nidulans* (Yong et al., 2010). Strikingly, all NRT2 proteins other than AtNRT2.1 were capable of mediating small levels of nitrate flux following the injection of the individual encoding mRNA sequences into Xenopus oocytes, with this being most apparent for *NRT2.4* and *NRT2.7* (Chopin et al., 2007a; Kiba et al., 2012; Kotur et al., 2012). Additional research focused on the specific mechanisms whereby NRT2 family proteins mediate high-affinity nitrate transport is thus warranted.

## 3 N starvation and remobilization

Under conditions of N deficiency, the NO_3_^-^ that is stored in plants can undergo remobilization and phloem-mediated transport (Wang et al., 2012). Marked increases in *NRT2.4* and *NRT2.5* expression are evident in response to N deprivation, with *NRT2.5* being induced at much higher levels than *NART2.4* in shoots and roots. Both of these genes are expressed in shoot vascular tissue in N-starved plants (Kiba et al., 2012; Lezhneva et al., 2014). In experiment in which plants were growth with access to normal N levels for 6 weeks followed by a 4-week period of N starvation, a 45% reduction in leaf phloem exudate NO_3_^-^ concentrations was observed in *nrt2.4* mutants relative to wild-type plants, without any corresponding change in *nrt2.5* mutants, and an even stronger reduction in *nrt2.4 nrt2.5* double mutants such that these exudate levels were just 20% of those observed in wild-type plants. This phenotype was restricted to phloem exudate NO_3_^-^ levels, as none of these mutants exhibited changes in whole leaf NO_3_^-^ concentrations or phloem exudate amino acid content, demonstrating specific roes for *NRT2.4* and *NRT2.5* in the remobilization of nitrate within shoots in response to N starvation (Kiba et al., 2012; Lezhneva et al., 2014).

**Figure 1:**
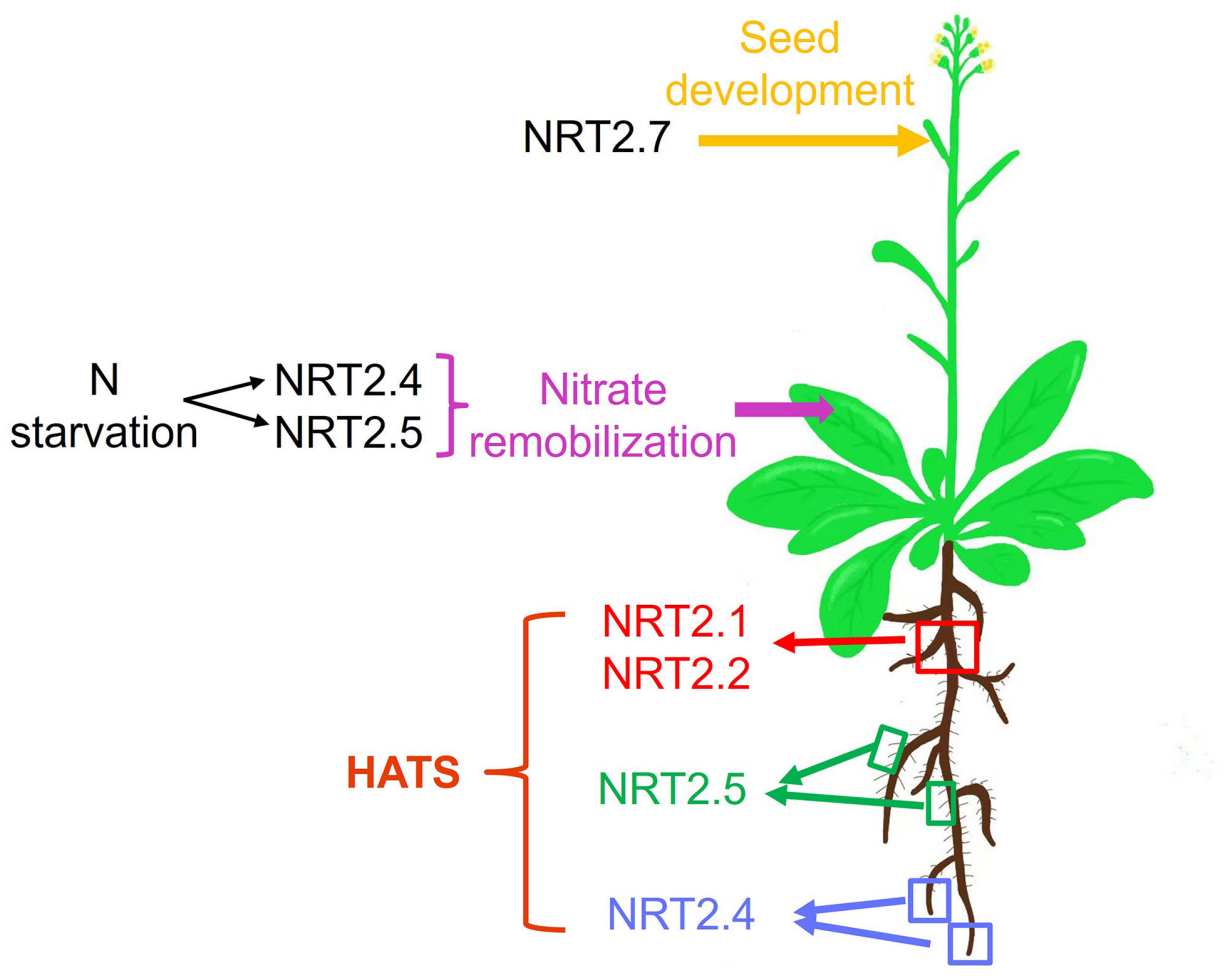
The physiological roles played by *Arabidopsis* NRT2 nitrate transporters, from the uptake of soil nitrate to its remobilization among leaves and the function of these transporters during seed development. HATS: high-affinity transport system; red square indicates the older parts of both primary roots and lateral roots; green squares indicate root hair of both primary roots and lateral roots; blue squares indicate the younger parts of both primary roots and lateral roots.

A summary of the various contributions of different NRT2 family members to specific phases of the processes of nitrate uptake and allocation is presented in **Figure 1** and **Table 1**. In *Arabidopsis*, NRT2.1, NRT2.2, NRT2.4, and NRT2.5 all serve as mediators of high-affinity nitrate uptake, although the functions of the latter two of these proteins are only evident in the context of N starvation. After an extended starvation interval, NRT2.5 expression levels are increased such that it serves as the primary high-affinity uptake transporter protein. *NRT2.1*, *NRT2.4*, and *NRT2.5* also exhibit differences in their spatial expression profiles, with *NRT2.1* expression primarily being evident in older portions of primary roots (Nazoa et al., 2003), whereas *NRT2.4* is most prominently expressed in younger portions of the primary roots and distal areas of lateral roots (Kiba et al., 2012), and *NRT2.5* is expressed in the root hair regions of both primary and lateral roots (**Figure 1**). Future research is warranted to clarify the degree to which nitrate affinity differs among these transporters, given that higher-affinity transporters may be important to allow plants to better deal with the stress associated with extended N starvation in soil with poor fertility. NRT2.4 and NRT2.5 expression are also evident in the phloem of the major and minor shoot veins, influencing shoot phloem nitrate levels under certain conditions or in the context of specific mutations. NRT2.7 expression is primarily evident during seed development in the tonoplast, wherein it serves to regulate seed nitrate levels.

## 4 Plant growth and development

### 4.1 Shoot growth

Members of the NRT2 family are vital to the activity of the NO_3_^-^ HATS (Cerezo et al., 2001; Filleur et al., 2001; Li et al., 2007), and they thereby strongly influence plant growth when cultivating plants in the context of NO_3_^–^poor solution (Orsel et al., 2004; Li et al., 2007; Kiba et al., 2012; Lezhneva et al., 2014). Indeed, *nrt2.1 nrt2.2* double mutant plants exhibit markedly reduced shoot growth as compared to wild-type plants, with their leaves turning a pink color (Li et al., 2007). The growth of *Arabidopsis* plants for 4 weeks in hydroponic tanks in a 1 mM NH_4_NO_3_ solution with subsequent nitrogen starvation for 1 week resulted in significantly lower shoot-to-root ratios and shoot weight for *nrt2.1* mutants as compared with wild-type plants, while no corresponding changes were evident for *nrt2.2* single mutants, and even greater reductions in both parameters were evident for *nrt2.1 nrt2.2* double mutants relative to plants in which only *nrt2.1* was mutated (Li et al., 2007). This supports a model wherein both of these genes serve as important regulators of growth, albeit with *NRT2.1* playing the most important role in this context. While no significant differences in *nrt2.4* single mutant plant fresh weight were detected when compared to wild-type controls cultivated in the presence of low (0.05 or 0.5 mM) KNO_3_ levels, the triple *nrt2.1 nrt2.2 nrt2.4* mutation was associated with even greater reductions in biomass productivity relative to *nrt2.1 nrt2.2* mutants, particularly at the lower tested KNO_3_ level. This may be attributable to the ability of NRT2.4 to mediate the high-affinity transport of nitrate only when its concentrations are very low (Kiba et al., 2012). In a similar vein, *nrt2.5* mutants and wild-type *Arabidopsis* exhibited comparable shoot biomass, while a 10% drop in shoot fresh weight was detected for triple *nrt2.1 nrt2.2 nrt2.5* mutant plants, and this decline was even more marked if *nrt2.4* was also mutated (Lezhneva et al., 2014). Together, these data provide clear evidence for the essential roles that NRT2.1, NRT2.2, NRT2.4, and NRT2.5 play in supporting plant growth when N levels are limited. This is consistent with the fact that the expression of these four genes is largely restricted to the roots, wherein they facilitate root NO_3_^-^ influx (Filleur and Daniel-Vedele, 1999; Orsel et al., 2002; Okamoto et al., 2003; Vidal et al., 2020). Rightfully so, mutation of the partner protein NRT3.1 resulted in the poor shoot growth when plant grown on plates containing 250 mM NO_3_^-^ as the sole nitrogen source (Okamoto et al., 2006). Shoot growth deficiencies in these mutants may thus be attributable to the long-distance effects of NO_3_^-^, indicative of the shifts in whole-plant N distributions.

### 4.2 Root system architecture

In order to contend with shifting soil N source availability under changing environmental conditions, plants have evolved a range of adaptive strategies that include root system architecture plasticity (Robinson, 1994; Zhang and Forde, 2000). Both nitrate and sugar can induce the expression of *NRT2.1* (Lejay et al., 1999; Cerezo et al., 2001). Relative to the use of standard growth medium, cultivating seedlings on media with a high C/N ratio results in the significant repression of lateral root initiation compared to a standard growth medium (Malamy and Ryan, 2001), whereas this repression was not evident in *nrt2.1* mutant plants (Little et al., 2005), supporting a role for *NRT2.1* in this repressive mechanism. Under nitrate-free conditions, such repression of lateral root initiation was still evident, simulating defective transport activity and thus revealing that this impairment of lateral root initiation was not nitrogen uptake-dependent (Remans et al., 2006b). NRT2.1 may thus serve as a sensor or signal transducer for nitrate involved in a signaling pathway that ultimately represses lateral root initiation. Relative to wild-type plants, those harboring *nrt2.1* mutations also presented with lower levels of lateral root growth following transfer from nitrate-rich to nitrate-poor medium (10 to 0.5 mM) (Remans et al., 2006b; Li et al., 2007), with this reduction being even more pronounced in *nrt2.1 nrt2.2* double mutants consistent with both of these genes serving as important factors involved in the regulation of lateral root growth (Li et al., 2007). NRT2.1 thus appears to help coordinate the development of lateral roots when NO_3_^-^ availability is limited.

Nitrate treatment can reportedly enhance the initiation and emergence of lateral roots (Vidal et al., 2010, 2013), with both of these processes being impaired for *nrt2.1 nrt2.2* mutant plants relative to wild-type controls cultivated in the presence of 1 mM ammonium for 2 weeks in a hydroponic system followed by a 3-day treatment with 5 mM KNO_3_. Strikingly, *tga1 tga4* and *nrt2.1 nrt2.2* plants presented with similar lateral root initiation phenotypes (Alvarez et al., 2014). In subsequent analyses, the TGA1/TGA4 transcription factors were identified as direct regulators of *NRT2.1/NRT2.2* (Alvarez et al., 2014), supporting their ability to regulate the development of lateral roots at least in part through the control of *NRT2.1* and *NRT2.2* expression.

A recent forward genetic screening effort additionally established NRT2.1 as a key regulator of primary root elongation under limited NO_3_^-^ stress conditions, as evidenced by the significant increase in primary root length for *nrt2.1* mutant seedlings cultivated in the presence of 0.05 mM NO_3_^-^ relative to wild-type controls. The root tips of these *nrt2.1* seedlings also exhibited higher levels of the key root growth regulator auxin as compared to wild-type root tips in the presence of low nitrate concentrations. However, *nrt2.1 pin7* double mutants exhibited root tips comparable to those of wild-type plants and shorter than those of *nrt2.1* plants, consistent with the ability of PIN7, which is an auxin efflux carrier, to function downstream of NRT2.1 as a regulator of the growth of roots in the presence of limited NO_3_^-^ availability. A series of assays confirmed the ability of PIN7 and NRT2.1 to physically interact with one another when NO_3_^-^ levels are low, thereby suppressing the PIN7-mediated acropetal efflux of auxin, thus slowing the elongation of primary roots. Together these results support a model in which NRT2.1 is capable of influencing root growth activity through interactions with the PIN7-mediated auxin transport machinery when levels of available NO_3_^-^ are low (Wang et al., 2023).

### 4.3 Seed development and germination

There are three primary stages to the process of *Arabidopsis* seed development. After initial morphogenesis, a maturation phase occurs that entails the production of N and C storage compounds in the form of seed storage proteins (Heath et al., 1986; Baud et al., 2002). NPF2.12/NRT1.6 localizes to the plasma membrane and is vital for early embryonic development (Almagro et al., 2008), with mutations in this gene reducing rates of nitrate accumulation within mature seeds while enhancing seed abortion rates. *NPF2.12* expression was only detectable in funiculus and silique vascular tissues, with upregulation immediately following pollination. This suggests a role for *NPF2.12* in the delivery of nitrate from maternal tissues to nascent embryos (Almagro et al., 2008). In contrast, NRT2.7 is primarily active within mature seeds. The homology of NRT2.7 is markedly lower relative to other NRT2 family members, sharing just 55% similarity with NRT2.1, for example (Orsel et al., 2002; Chopin et al., 2007a). Unlike most other members of this gene family, it is also primarily expressed in developing seeds rather than in roots, with its upregulation being particularly pronounced as seeds undergo dessication (Orsel et al., 2002; Okamoto et al., 2003; Chopin et al., 2007a). While oocyte-based experiments have confirmed that NRT2.7 can function as a nitrate transporter, it has no role in the direct uptake of soil nitrate via the roots, nor does it impact the distribution of nitrate in plant vegetative organs (Chopin et al., 2007a). Subcellular localization analyses have demonstrated that NRT2.7 primarily localizes to the tonoplast surrounding the vacuoles. Studies of the effects of *nrt2.7* mutations on seeds have been conducted with the mutant *nrt2.7-1* (Col-8 background) and *nrt2.7-2* (Ws background) plant lines. Both exhibit similar seed weights to those of wild-type plants, but reduced seed nitrate levels under nonlimiting N conditions. Nitrate has also been posited to serve as a signal that can trigger seeds to break dormancy and begin germination (Alboresi et al., 2005; Chopin et al., 2007a). In line with such a model, when the same batches of freshly harvested seeds were sown on water-containing medium, both *nrt2.7* mutants exhibited germination delays relative to wild-type controls within 2 days. While *nrt2.7-2* mutants exhibited lower rates of germination throughout a 7-day analytical period relative to the control Ws line, no apparent difference in germination was evident between the Col-8 and *nrt2.7-1* mutant lines from days 3-7 post-sowing (Chopin et al., 2007a). Col seeds and foliar tissues exhibited higher nitrate storage capabilities relative to those of Ws plants, suggesting that Col plants are better able to tolerate N deprivation (Chopin et al., 2007a; North et al., 2009). Differences in such tolerance among plant ecotypes may thus account for varying seed germination phenotypes. Overall, these data highlight a key role for NRT2.7 in seed nitrate concentration and germination.

David et al. performed further characterization of *nrt2.7-2* mutants exhibiting a distinctive phenotype consisting of a seed coat that was a plane-brown color in contrast to that of wild-type Ws (David et al., 2014). Seed coloration is generally related to flavonoid oxidation levels (Pourcel et al., 2005; Lepiniec et al., 2006; Routaboul et al., 2012), and additional analysis indicated that these *nrt2.7-2* mutant seeds accumulated higher levels of soluble proanthocyanidins (PAs) that could undergo oxidation in the testa with seed dessication (David et al., 2014). This seed PA accumulation was apparently unrelated to fluctuations in seed NO_3_^-^ content, in line with the observation that *npf2.12* and *clca* mutant seeds did not exhibit any change in color or PA content despite the reduction in NO_3_^-^ levels therein (Almagro et al., 2008; Monachello et al., 2009). These data support a specific link between the accumulation of PA in seeds and the function of NRT2.7, rather than linking it to NO_3_^-^ accumulation. Lower NO_3_^-^ levels and higher concentrations of soluble PAs were also apparent in *nrt2.7-2* mutant seeds relative to Ws, resembling *tt10* mutant phenotypes (David et al., 2014). The TRANSPARENT TESTA 10 (TT10) protein serves as a laccase candidate enzyme that facilitates the oxidative polymerization of PAs and other flavonoids (Pourcel et al., 2005). No studies to date, however, have revealed any ability of NRT2.7 to influence the enzymatic activity of TT10, and additional research aimed at clarifying the activity of TT10 will be vital to understanding the mechanisms that ultimately result in the higher levels of soluble Pas within *nrt2.7-2* seeds. These findings thus reveal a central role for NRT2.7 as a regulator of the accumulation and oxidation of PAs within seeds. While NRT1 family proteins have been shown to serve as transporters for non-nitrate molecules (Léran et al., 2014), whether NRT2 proteins can function in a similar manner remains poorly understood, and additional research will be vital to test this hypothesis.

## 5 *AtNRT2.1, AtNRT2.5*, and *AtNRT2.6* are influence plant-microbe interactions

Plant nutritional status can strongly shape the ability of these plants to defend against pathogens such as *Pseudomonas syringae* (Long et al., 2000; Modolo et al., 2005, 2006). Relative to wild-type controls, *nrt2.1* and *nrt2.1 nrt2.2* mutant plants exhibit a reduction in susceptibility to *P. syringae pv tomato DC3000* (*Pst*) (Camañes et al., 2012). Under infection conditions, *nrt2.1* exhibited more robust and more rapid SA-dependent defense priming, which was a key mechanism responsible for enhanced *Pst* resistance (Zimmerli et al., 2000; Conrath et al., 2006; Jung et al., 2009). These *nrt2.1* mutants were also partially deficient in their ability to detect coronatine, a bacterial effector important in the context of infection (Brooks et al., 2004; Melotto et al., 2008; Camañes et al., 2012). These decreases in *nrt2.1* susceptibility to *Pst* may thus stem from both coronatine insensitivity and improved SA priming. The inoculation of plants with the phytopathogen *Erwinia amylovora* also resulted in an increase in the expression of *NRT2.6*, with plants expressing lower *NRT2.6* levels exhibiting greater pathogen susceptibility as a consequence of impaired reactive oxygen species production, although these *nrt2.6* mutants did not exhibit any apparent nitrate-associated phenotypes (Dechorgnat et al., 2012). Together, these data suggest that members of the NRT2 family can serve as sensors for a range of environmental stimuli, thereby coordinating abiotic and biotic stress responses in addition to shaping the ability of plants to respond to nutritional cues.

The plant growth-promoting rhizobacterium (PGPR) strain *Phyllobacterium brassicacearum* STM196 has been reported to promote the growth of *Arabidopsis* and to overcome lateral root developmental inhibition under conditions of high nitrate availability (Mantelin et al., 2006). Notable increases in *NRT2.5* and *NRT2.6* expression have been observed in plants exposed to STM196, but *nrt2.5* and *nrt2.6* mutants failed to exhibit such STM196-induced growth (Kechid et al., 2013), indicating that these two genes encode proteins that can influence the outcomes of beneficial biotic interactions.

## 6 Biological processes by *AtNRT2.1*

### 6.1 AtNRT2.1 controls cadmium uptake

Supplying plants with NO_3_^-^ has been shown to result in higher concentrations of Cd and more pronounced Cd toxicity in exposed plants (Mao et al., 2014; Yang et al., 2015; Cheng et al., 2020). Moreover, nitrate transporters NPF6.3/NRT1.1, NPF7.3/NRT1.5, and NPF7.2/NRT1.8 are responsive to Cd stress conditions in *Arabidopsis*, regulating the accumulation of Cd under conditions of both high and normal NO_3_^-^ availability (Li et al., 2010; Chen et al., 2012; Mao et al., 2014; Wang et al., 2018). Further studies have indicated that Cd can suppress the expression of key HATS-related genes including *NRT2.1*, *NRT2.2*, and *NRT2.4*, thereby suppressing the uptake and accumulation of nitrate in roots when nitrate levels are low, which results in a corresponding reduction in root Cd uptake (Guan et al., 2021). This suggests that efforts to control nitrate transporter activity may provide a means of abrogating Cd accumulation when growing crops in soil contaminated with this heavy metal.

### 6.2 AtNRT2.1 influences light-responsive carbon and nitrogen metabolism

As reported previously, root transport systems are generally regulated by shoot photosynthetic activity, especially in the context of the uptake of NO_3_^-^ (Delhon et al., 1995; Forde, 2002). NO_3_^-^ uptake is under the control of downwardly transported sugars, CO_2_, carboxylic acids, and other photosynthates (Delhon et al., 1996). Root *NRT2.1* expression has been demonstrated to be both sugar- and light-inducible (Lejay et al., 1999). However, sugar analog treatment or analyses of sugar-sensing mutant plants revealed no changes in sugar-induced *NRT2.1* induction, suggesting that this process occurs through a mechanism distinct from the primary mechanisms that have been documented to facilitate plant sugar sensing (Lejay et al., 2003). Mutants lacking the expression of hexokinase (HXK), in contrast, exhibited an absence of sugar-induced *NRT2.1* expression, consistent with the metabolic activity downstream of HXK being key to this regulatory process, rather than sugar itself (Lejay et al., 2003). *HXK* catalyzes a reaction that produces glucose-6-P (G6P), and treating roots with glycerol to reduce G6P concentrations can strongly impair normal *NRT2.1* upregulation following the dark/light transition (Lejay et al., 2008). However, the treatment of plants with 6-aminonicotinamide (6-AN), which can potently inhibit phosphor-gluconate dehydrogenase activity and impair OPPP, the near total absence of sugar-induced *NRT2.1* expression was evident despite no corresponding change in G6P levels relative to sucrose treatment. This suggests that *NRT2.1* upregulation in response to C signals is associated with OPPP activity rather than being directly induced by G6P (Lejay et al., 2008). Relative to wild-type plants, *gin2-1* mutant plants with defective sugar responses exhibited impaired *NRT2.1* upregulation in response to Glc, while treatment with the OPPP intermediates shikimate and pyruvate was sufficient to restore this defect (de Jong et al., 2014). Sugar-induced *NRT2.1* expression is thus dependent on the OPPP pathway.

C and N acquisition rates are regulated in a tightly coupled manner (Matt et al., 2010; Nunes-Nesi et al., 2010), and light serves an important regulatory role for both of these processes (Lillo, 2008). Wild-type *Arabidopsis* seedlings with shoots and roots respectively exposed to light and dark conditions [S(L)/R(D)] exhibited significant increases in both primary root length and NO_3_^-^ uptake as compared to wild-type seedlings placed under the opposite conditions [S(D)/R(L)], supporting a model wherein light-induced shoot-to-root signaling activity can favor nitrate uptake and the growth of roots (Chen X. et al., 2016). Strikingly, mutations in the *HY5* gene encoding a photomorphogenic bZIP transcription factor were capable of eliminating this nitrate uptake and root growth induced by shoot illumination, and further hypocotyl graft chimera-based studies codified HY5 as a shoot-root phloem-mobile signal (Chen X. et al., 2016). The *nrt2.1* mutant plants also exhibited reduced levels of NO_3_^-^ uptake in response to shoot illumination, with HY5 derived from shoot tissue promoting the autoactivation of HY5 in the roots, thereby promoting NO_3_^-^ uptake in the roots via *NRT2.1* activation (Chen X. et al., 2016). *NRT2.1* promoter binding by HY5 can be enhanced when sucrose availability, with HY5 regulating its fixation and translocation (Chen X. et al., 2016). Mobile HY5 thus serves as an important regulator of *NRT2.1* in the context of illumination-responsive N and C metabolism.

### 6.3 AtNRT2.1 involves in systemic nitrate signaling mechanisms

**Figure 2:**
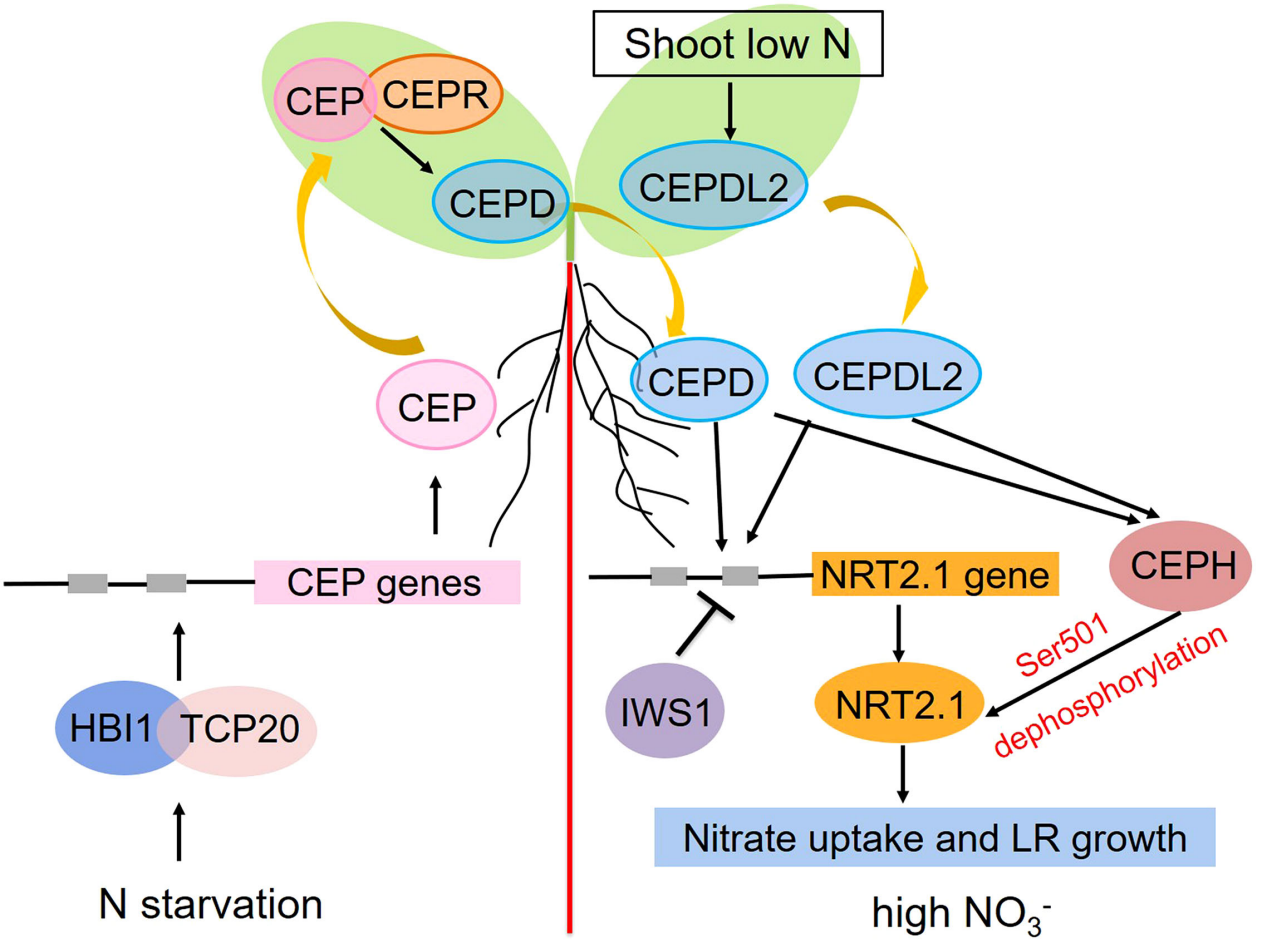
Schematic overview of the roles that *AtNRT2.1* plays in the systemic nitrate signaling pathway under heterogeneous conditions. Positive and negative regulation are respectively represented with arrows and blunted lines.

Soil nitrate distributions are generally heterogeneous. To adapt to this inconsistent availability, plants have evolved intricate systemic responses whereby stimuli that are perceived at the local level can be communicated to distant organs. For example, in plants grown in split-root plates for which half of their root system was nitrate-deprived while the other half was in a nitrate-rich environment, more pronounced proliferation of roots on the nitrate-rich side was evident relative to plants cultivated under homogenously nitrate-rich conditions, with this response being dependent on shoot nitrate accumulation (Remans et al., 2006a; Ruffel et al., 2011; Vidal et al., 2020). The transcription factor gene *TEOSINTE BRANCHED1/CYCLOIDEA/PROLIFERATING CELL FACTOR1-20 (TCP20)* mutations in these split-root experiments were sufficient to impair the preferential growth of lateral roots on the nitrate-rich site (Guan et al., 2014). Additional analyses demonstrated the ability of TCP20 to interact directly with HOMOLOG OF BRASSINOSTEROID ENHANCED EXPRESSION2 INTERACTING WITH IBH1 (HBI1), a bHLH transcription factor, with nitrate starvation enhancing this interaction. The resultant HBI1-TCP20 complex was then capable of directly binding the C-terminally encoded peptide (CEP) promoters, inducing their expression in a cooperative fashion (Chu et al., 2021). The resultant CEPs were secreted from roots and functioned as ascending signals of N starvation that were detected by the LRR-RK receptor (CEPR) in shoots, thereby triggering shoot-derived descending CEPD1 (CEP downstream 1) and CEPD2 peptide production. These peptides, in turn, triggered the upregulation of *NRT2.1*, *NRT3.1*, and other nitrate-related genes in the roots, thereby inducing higher levels of nitrate uptake and root proliferation in areas rich in nitrates (Tabata et al., 2014; Ohkubo et al., 2017). The CEPD-like 2 (CEPDL2) peptide was also induced by low nitrate concentrations and shoot N deprivation in a CEPR-independent fashion, whereupon it functions as a leaf-derived systemic signal that controls the root-mediated uptake of nitrate by regulating the expression of key genes including *NRT2.1, NRT3.1*, and *NRT2.4* (Ota et al., 2020). The RNAPII complex component IWS1 is also capable of suppressing the expression of *NRT2.1* under high levels of nitrate availability by enhancing the H3K27me3 of the chromatin region encoding this gene (Girin et al., 2010; Widiez et al., 2011). A range of post-translational mechanisms also shape the activity of NRT2.1 in response to systemic N-related signaling activity. For example, the CEPD1/2- and CEPDL2-mediated signals are capable of promoting the upregulation of CEPH, a root-specific PP2C phosphatase that can dephosphorylate NRT2.1 Ser501 to activate the high-affinity uptake of nitrate in *Arabidopsis* (Ohkubo et al., 2021). At the systemic level, both transcriptional and post-translational switches govern the activity of NRT2.1 to maintain N homeostasis, particularly under conditions of limited soil N availability and/or increased shoot N demands (**Figure 2**).

## 7 Conclusions

Since the first identification of *crnA* as the founding member of the *NRT2* gene family, countless studies have sought to clarify the characteristics and functions of different NRT2 proteins in specific plant species. This review offers an overview of the spatiotemporal expression patterns, localization, biotic and abiotic responses, and functional roles of the seven *AtNRT2* genes (**Table 1**). While some progress has been made on this front to date, much remains to be done. For example, how *AtNRT2.3* and *AtNRT2.6* contribute to nitrate-related regulatory processes remains uncertain, and it remains uncertain as to whether NRT2 proteins can serve as transporters for molecules other than nitrate. Similarly, the status of NRT2.1 as a potential nitrate sensor warrants further investigation. As such, additional studies will be vital to fully clarify how plants are able to sense and respond to changing environmental conditions in order to provide a sound basis for crop-focused research.

*Arabidopsis-*based findings can be extended to other economically important plant species, highlighting valuable targets for efforts to enhance crop yields in harsh or otherwise unfavorable environments. In recent years, significant progress has been made in the research of *NRT2* genes in crops. In rice, overexpression of *OsNRT2.1* increased the transcription level of auxin transporter genes *OsPIN1a/b/c* and *OsPIN2* and which in turn promoted total root length under 0.5 mM NO_3_^-^ conditions (Naz et al., 2019). Knockout of *OsNRT2.4* inhibited lateral root length and number under 0.25 Mm and 2.5 mM NO_3_^-^ conditions (Wei et al., 2018). In addition, knockdown of *OsNAR2.1* suppressed lateral root formation under low NO_3_^-^ conditions (Song et al., 2020). These results suggest that *OsNRT2.1/NRT2.4/NAR2.1* play a critical role in controlling root development. Moreover, transgenic lines overexpressing *OsNRT2.1/2.2* could improve nitrogen use efficiency (NUE) and grain yield in rice (Chen J. et al., 2016; Hu et al., 2023). Transgenic lines co-overexpressing *OsNAR2.1* and *OsNRT2.3a* increased agronomic nitrogen use efficiency (Chen et al., 2020). Meanwhile, Fan’s group reported that overexpression of *OsNRT2.3b* could also enhance NUE and rice grain yield in the field (Fan et al., 2016). In wheat, transcription factor TaNAC2-5A could directly bind to the promoters of *TaNRT2.1-6B*, *TaNRT2.5-3B*, *TaNPF7.1-6D*, and *TaGS2-2A* and activate their expression to affect NO_3_^-^ transport and assimilation, and ultimately increased grain yield and NUE (Li et al., 2020; Gao et al., 2022). Furthermore, new information have been obtained about unexpected peculiar roles played by some *NRT2* genes. The NRT2 transporter family has at least 4 members in *Lotus japonicus* (Criscuolo et al., 2012), 5 in *Oryza sativa* (Feng et al., 2011b) and 49 in the *Wheat* genome (Deng et al., 2023). In *Lotus japonicus*, high NO_3_^-^ treatment attenuated nodulation, but the effect of nitrate was suppressed by the *LjNRT2.1* mutation (Misawa et al., 2022). Of note, the nodulation phenotypes of the *Ljnlp1* and the *Ljnlp4* mutants are similar to those of the *Ljnrt2.1* mutants under high NO_3_^-^ conditions (Nishida et al., 2021). Further investigation suggested that NODULE INCEPTION (NIN)-LIKE PROTEIN1 (*LjNLP1*) could directly bind to the *LjNRT2.1* promoter and activate its transcript and subsequently promote nitrate uptake/transport process, which ultimately affected nuclear localization of LjNLP4 and subsequent regulation of the expression of nodulation-related genes (Misawa et al., 2022). Phylogenic analysis revealed *LjNRT2.4* to be a close relative of *AtNRT2.7* which was the most diverged of all the NRT2 sequence (Valkov et al., 2020). Unlike *AtNRT2.7* which expressed mainly in seeds and the protein localized to vacuolar membrane (Chopin et al., 2007a), *LjNRT2.4* was expressed in root and nitrogen-fixing nodule vascular tissues and localized at the plasma membrane. Mutation of *LjNRT2.4* caused much more severe N_2_-fixation related phenotypes in nodulated plants grown under hydroponic conditions (Valkov et al., 2020). In rice, all OsNRT2 members except OsNRT2.4 which shares the highest value of amino acid identity with AtNRT2.7 need OsNAR2.1 for root NO_3_^-^ acquisition in response to both low- and high- nitrate supply (Wei et al., 2018). Interestingly, unlike other NRT2s which function as the high-affinity NO_3_^-^ transporter, OsNRT2.4 is a dual-affinity NO_3_^-^ transporter (Wei et al., 2018). These findings are just the tip of the iceberg, more endeavors are needed to decipher the mechanism of NRT2 family, improve NUE in crops, eliminate the pollution from N as field fertilizer, and maintain nutrient homeostasis.

## Author contributions

NX: Writing – original draft. LC: Writing – original draft. YK: Writing – original draft. GC: Writing – review & editing. LZ: Writing – review & editing. FL: Writing – review & editing.
